# Numerical and Experimental Analysis of the Load-Carrying Capacity of a Timber Semi-Rigid Dowel-Type Connection

**DOI:** 10.3390/ma15207222

**Published:** 2022-10-17

**Authors:** Marek Johanides, Antonin Lokaj, Pavel Dobes, David Mikolasek

**Affiliations:** 1 Department of Structures, Faculty of Civil Engineering, VSB-Technical University of Ostrava, 708 00 Ostrava, Czech Republic; 2 Department Centre of Building Experiments, Faculty of Civil Engineering, VSB-Technical University of Ostrava, 708 00 Ostrava, Czech Republic

**Keywords:** bolts and dowels, dowel-type fasteners, FEM, frame connection, fully threaded screws, glued laminated timber, numerical model, rotational stiffness

## Abstract

The paper deals with the analysis of the load-carrying capacity of a timber semi-rigid connection created from a system of two stands and a rung. The connection was made from glued laminated timber with metal mechanical dowel-type fasteners. Not only a common combination of bolts and dowels, but also fully threaded screws were used for the connection. The aim of the research and its motivation was to replace these commonly used fasteners with more modern ones, to shorten and simplify the assembly time, and to improve the load-carrying capacity of this type of connection. Each of these two types of connections was loaded statically, with a slow increase in force until failure. The paper presents results of the experimental testing. Three specimens were made and tested for each type of the connection. Experimental results were subsequently compared with numerical models. The achieved results were also compared with the assumption according to the currently valid standard. The results indicate that a connection using fully threaded screws provides a better load-carrying capacity.

## 1. Introduction

Since the mid-1950s, many countries have carried out extensive research in the area of wood-based materials and their fasteners. Special regulations for construction procedures were necessary in some countries because of the different species of timber. This led to various theories, which resulted in even more significant differences in design criteria. The progress in timber engineering design soon showed the need for more extensive research in the behavior of timber structures, including their connections. Experimental tests made in Forest Products Laboratory *Wilson 1917* [1] were among the first instances of research in the area of nailed connections.

The current model for the determination of the load-carrying capacity of dowel-type connections under a lateral load is the European yield model, abbreviated as EYM. The EYM theory arose from the research of the Danish scientist *Johansen 1941* [2], when he first applied this theory to timber fasteners. Eight years later, *Johansen 1949* [3] published an original article about EYM in English. Some of his research remained unfinished, and the results were unpublished for years. Finally, this research was completed and published by *Larsen 1977* [4]. The theory was based on, and later experimentally verified on single-shear and double-shear connections with one nail by *Möller 1951* [5]. Other researchers followed up on the groundbreaking research in the area of the design of connections in timber structures. American scientist *Kuenzi 1955* [6] dealt with a permissible transverse load of single-shear and double-shear nailed and bolted connections. The scientific articles by Johansen and Möller were complemented with the load–deformation response of nailed and bolted connections. It was found that the plastic deformation of a fastener causes non-linearity and contributes significantly to the load-carrying capacity of the connection. He presented the idea of a fastener as a beam on an elastic foundation (supported by so-called Winkler springs; see *Winkler 1867* [7]). The derived relations were valid for the elastic branch of the load–deformation curve of the connection. However, they became the basis for further research in the modeling of dowel-type connections using nonlinear parameters for embedment in timber; for example, the ariticle by *Foschi 2000* [8].

*Meyer 1957* [9] tested nailed connections with different qualities of timber and nails, and proved that the load-carrying capacity of the connection is significantly influenced by the thickness and the embedment strength of the used timber. *Doyle 1964* [10] observed that there is a dependence between the load-carrying capacity and the end distance, the spacings parallel to the grain, and the fastener diameter. *Mack 1966* [11] created an analytical model for transversely loaded nailed connections, where the load–deformation response to short-term loading is influenced by many mutual independent factors. *Larsen and Reestrup 1969* [12] investigated screwed connections and found that the conditions in a transversely loaded screwed connection differ slightly from a bolted connection due to the thread of the screw. This meant that different values of the fastener yield moment had to be considered. *Norén 1974* [13] stated different formulas for different types of connections in timber structures. *Larsen 1973* [14] proposed a supporting rationale for the EYM theory in the Scandinavian countries. Afterwards, *Larsen 1979* [15] proposed a theoretical background and approximations for screwed connections. The findings were later included into the *CIB Structural Timber Design Code 1983* [16]. The EYM model was further refined depending on the angle between the applied force and the grain direction; see *Smith and Whale 1986* [17]. It was proven that the direction of the applied force has no influence on the load-carrying capacity of connections with dowel-type fasteners up to a diameter of 8 mm. This work also became the basis for a standard relationship for the embedment strength. *Ehlbeck and Werner 1992* [18] followed up on this scientific article and investigated the load-carrying capacity of dowel-type connections depending on various selected parameters. *Blaß 2000* [19] dealt with the bending properties of dowel-type metal fasteners. He derived an empirical relationship that is used in the current standard for the calculation of the yield moment. Currently, the load-carrying capacity of connections is calculated on the basis of the relationships given in *Eurocode 5* [20]. This standard provides sufficient findings for determining the load-carrying capacity of transversely loaded connections in timber structures. However, the standard does not provide the procedure for calculating the load-carrying capacity of a connection loaded by the bending moment. Therefore, *Koželouh 1998* [21] was used to determine this load-carrying capacity.

Dowel-type fasteners, such as screws, bolts, and dowels, are one of the most popular fasteners in timber structures, not only in timber-to-timber connections—*Solarino 2017* [22] and *Vavrusova 2016* [23]—but also in steel-to-timber and aluminum-to-timber connections; see *Chybiński, Polus 2022* [24], *Chybiński, Polus 2022* [25]. In order to effectively apply dowel-type connections, it is crucial to understand their mechanical behavior under loading. It is desirable to know the relation between the load and slip, stress distribution, or possible different failure modes. The mechanical behavior of connections in timber structures is a complex problem that is influenced by a number of factors. The most important factors are the geometry and arrangement of the connection (i.e., spacings, edge, and end distances) as in *Cai 2019* [26], the material characteristics, the timber species as in *Požgaj 1986* [27], and the method of loading.

The load-carrying capacity and stiffness of connections are influenced by mechanical properties of fasteners and timber. The findings described in *Mirski 2019* [28] and *Mirski 2020* [29] can be used to determine the mechanical properties and to classify the structural timber. It is possible to use selected non-destructive experimental testing, as in *Nowak 2019* [30] and *Nowak 2021* [31]; dynamic testing, as in *Bragov 2020* [32]; and the non-destructive vibrational method described in *Olaoye 2021* [33] in order to determine these mechanical properties. A semi-destructive method can also be used to determine the density and moisture content of timber, as described in *Martínez 2020* [34].

Nowadays, numerical modeling is definitely an important part of experimental testing. It can be an excellent tool for the understanding of the behavior of connections in timber structures. *Dobeš 2022* [35], *Braun 2022* [36], and *Kupniewska 2021* [37] dealt with the calibration and validation of numerical models according to experimental tests.

This paper is focused on the experimental determination of the load-carrying capacity of a semi-rigid connection. The experimental tests are further validated by numerical models. The fist variant of the connection is made of a combination of bolts and dowels in Experiment A. The second variant of the connection is made of high-tensile fully threaded screws in Experiment B. The fasteners in Experiment A are commonly used in practice. Findings on the design of such a semi-rigid connection can be found in *Shu-Rong 2021* [38], *Mingqian 2019* [39], and *Wang 2021* [40]. On the other hand, the fasteners in Experiment B are not commonly used in practice. The presented paper is a follow-up research of *Johanides 2020* [41] and *Johanides 2021* [42]. These papers dealt with determining the rotational stiffness and load-carrying capacity of a timber frame corner, which was made of larger cross-sections with a greater number of fasteners. Small semi-rigid connections with the two already mentioned types of fasteners were subsequently created for a detailed analysis of the connection. The article by *Johanides 2022* [43] dealt with the determination and comparison of the ductility of this connection. The article by *Johanides 2022* [44] dealt with the determination and comparison of the rotational stiffness of this connection. The latest information from the series of tests is presented in this article, which deals with the determination of the load-carrying capacity.

The paper brings findings about the results of the analytical, experimental, and numerical determination of the load-carrying capacity of a semi-rigid connection with mechanical fasteners.

## 2. Materials and Methods

### 2.1. Description of Construction and Geometry

The tested specimens corresponded to actual connections used in practice. The arrangement and loading of test specimens was designed to correspond with the actual state of the connection in a real load-carrying structure. The air temperature was 21 °C and the relative air humidity was 55% during the experimental testing. The measurement was performed using TFA DC 106 device.

The structural system for the experiments was created from a semi-rigid connection of two stands and a rung. These structural elements were made of spruce timber, which is the most used structural timber in Central Europe for structural practice due to its availability. The disadvantage of this timber species is its low durability in the outdoor environment. The connection was made with dowel-type metal fasteners. Two identical structural systems were created with different types of fasteners. Experiment A contained a combination of bolts and dowels as fasteners. Experiment B contained fully threaded screws. Glued laminated timber of the strength class GL24h was used. Four-point bending tests were performed to verify the properties of the used timber. Results of the tests have already been published in *Johanides 2022* [43]. The stands were made of a cross-section of 100/300 mm and the rung was made of a cross-section of 100/300 mm. The material properties of the fasteners were also experimentally verified by tensile tests in *Johanides 2022* [43].

The fasteners used in experiment A consisted of bolts and dowels. These were created from a threaded rod with an outer diameter of 8 mm and a core diameter of 7.25 mm. The length of the bolt was 360 mm and the length of the dowels was 300 mm. The fasteners used in experiment B consisted of fully threaded screws with a diameter of 8 mm and a core diameter of 5 mm. Both used materials of fasteners were made of steel, grade 10.9. In addition, the holes for these fasteners were pre-drilled to minimize the initial slip of the connection.

**Figure 1:**
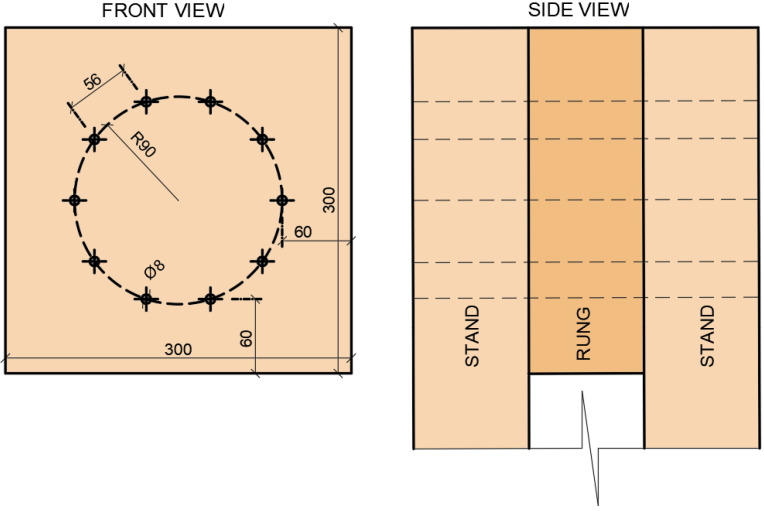
A location of fasteners in test specimens.

The arrangement was identical for both experiments. The fasteners were located on one symmetrical circle *r* = 90 mm, with 10 pieces. The arrangement (see Figure 1) was determined according to *Koželouh 1998* [21].

**Figure 2:**
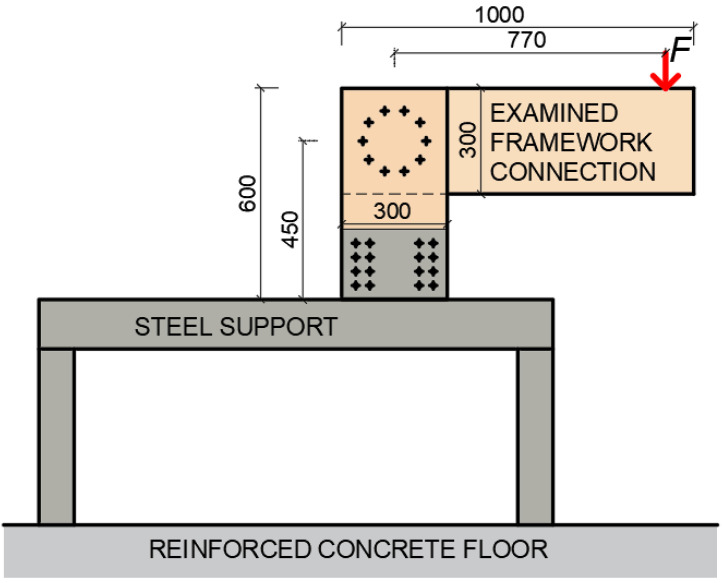
Schematic illustration of the investigated structural system.

The boundary conditions of the structure were created for the execution of the experiments. They were supported by using an auxiliary steel structure. This structure was designed and built for approximately three times the estimated applied force. Timber stands were then attached to the steel structure using 24 bolts with a diameter of 8 mm. The entire steel structure was placed on the reinforced concrete floor and centered at the required distance from the press head so that the press head pressed exactly at the desired location of the rung. After the correct setting of the steel structure, its rear part (Figure 2 on the left) was loaded with a steel cube of 1000 kg. This cube ensured the elimination of the tensile forces caused by the load. The right part did not need to be loaded in any way because only the compressive force was transmitted to this part of the structure. The schematic illustration of the experiment is shown in Figure 2.

### 2.2. Description of the Testing Machine

The experiments were performed using a LabTest 6.1200 electromechanical testing machine from *Labortech* (Opava, Czech Republic) [45] with a maximum force of 1200 kN. The testing machine is designed for tensile, compressive, static, and cyclic dynamic testing. The machine allows for testing using displacement controlled load. The testing speed varies from 0.0005 mm/min to 250 mm/min. The machine also allows for testing using force-controlled load up to 1200 kN, with a measurement accuracy of 1%. Control of the test device is provided by a PC with software.

### 2.3. Description of the Testing

The aim of the static testing was to investigate the total load-carrying capacities of the semi-rigid connection and the failure mode.

**Figure 3:**
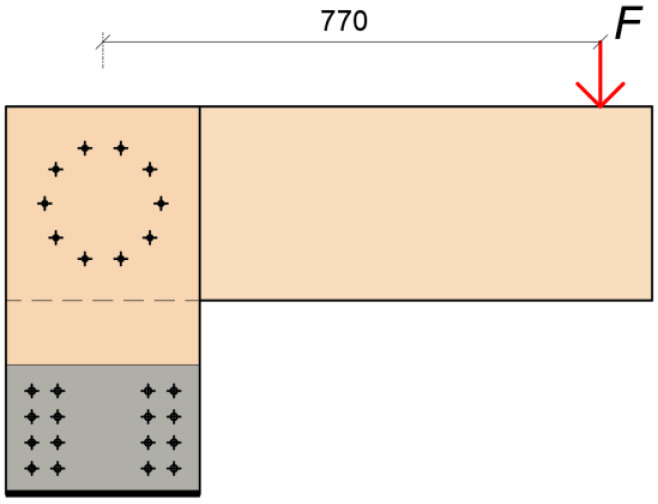
The position of the applied load and deformation measurement.

Specimens were subjected to a displacement-controlled loading (see Figure 3) after fastening to the auxiliary steel structure. The testing was aimed at determining the maximum load-carrying capacity, and therefore the deformation was measured by the displacement of the crosshead. The loading course was carried out in accordance with the standard *EN 26891* [46]. This standard specifies requirements and test methods for connections in timber structures using mechanical fasteners. The following loading procedure was prescribed:

1. Estimation of the maximum force F_est_ for the tested connection based on experience, calculation, or pretests;
2. Loading of the specimen to 40% of the estimated maximum force, 0.4·F_est_, then holding for 30 s;
3. Unloading to 10% of the estimated maximum force, 0.1·F_est_, then holding for 30 s;
4. Reloading until the specimen fails.

Table 1 shows the values that determine the course of the experimental loading for Experiment A (bolts and dowels) and Experiment B (fully threaded screws). The load-carrying capacity of the connection *F_ed_* was calculated as the design value (using modification factor *k_mod_* = 0.90 and partial factor for material properties *γ_c_* = 1.30). This value represents the maximum load-carrying capacity of the connection and was calculated according to *Eurocode 5* [20] and the literature *Koželouh 1998* [21].

The loading speed was chosen as constant in kN/min according to the selected loading schemes. The total testing time of one specimen was 15 min.

**Figure 4:**
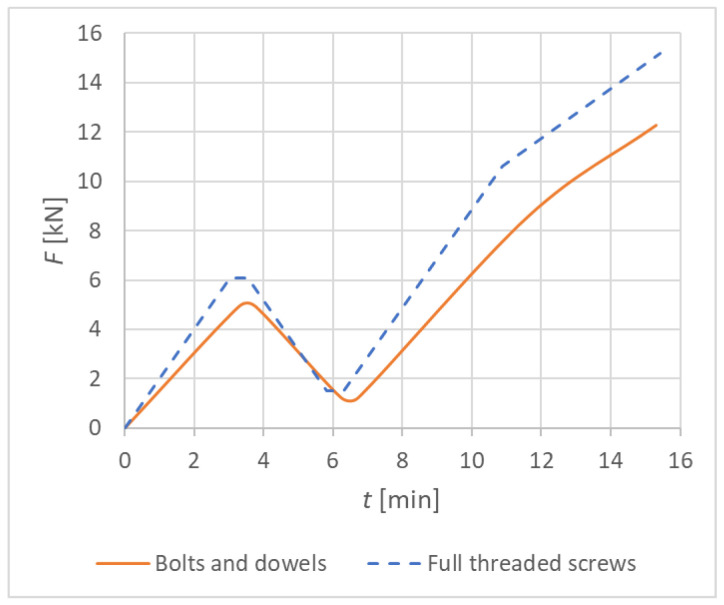
Course of the loading; see *Johanides 2022* [43].

Figure 4 shows the graphic course of individual experimental tests.

### 2.4. Experimental Testing

**Figure 5:**
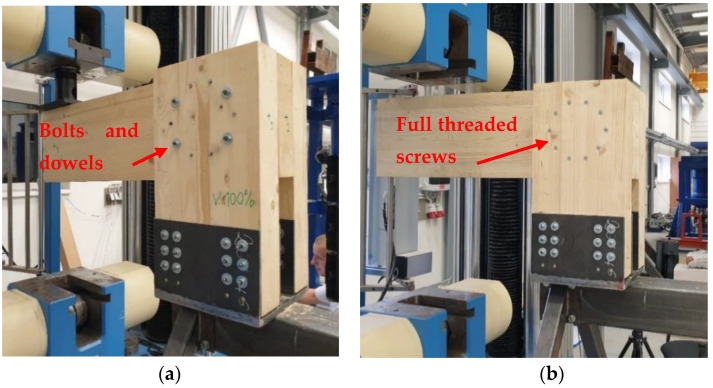
Experimental specimens: (**a**) Experiment A, bolts and dowels; (**b**) Experiment B, fully threaded screws; see *Johanides 2022* [43].

All of the experiments were carried out at the Centre for Building Experiments and Diagnostics at VSB—Technical University of Ostrava, Czech Republic. Figure 5a shows Experiment A, a combination of bolts and dowels. Figure 5b shows Experiment B, fully threaded screws. The load was applied to the connection using a steel cylinder with a diameter of 50 mm. A rubber pad with a thickness of 10 mm was placed under this cylinder to eliminate local damage of the timber rung during loading.

### 2.5. Numerical Modeling

**Figure 6:**
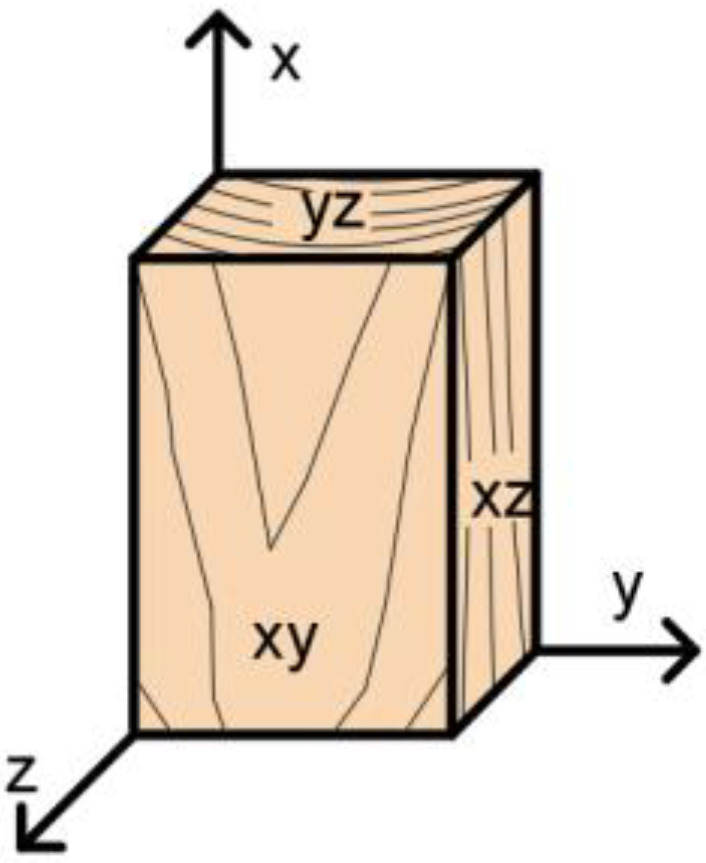
Orthotropy of timber.

*Ansys^TM^ 21* software in the *Workbench 21* environment [47] was used to create the numerical model. The geometry of the model was made up of 3D volume finite elements, which included physical, geometric nonlinearity, and contact elements. The material model of timber was considered orthotropic (see Figure 6) with plastic behavior. The condition of plasticity was created by Hill in *Brožovský 2012* [48]. Neglecting the orthotropy of timber would result in an insufficient representation of the behavior. The dissertation of *Mikolášek 2012* [49] and the publication *Gunderson and Goodman 1973* [50] were used to select the parameters of the material characteristics.

The material characteristics of timber are shown in Table 2, and the material characteristics of steel are shown in Table 3. The modulus of elasticity of fasteners was determined on the basis of the experience of the authors.

The values of the plastic behavior of timber (see Table 4) and the plastic behavior of steel (see Table 5) were obtained based on an experimental testing. The results of the testing have already been published in [43].

The Hill yield criterion allows us to set a single value for the hardening modulus of all directions. It was set to 10 MPa for a good numerical convergence.

**Figure 7:**
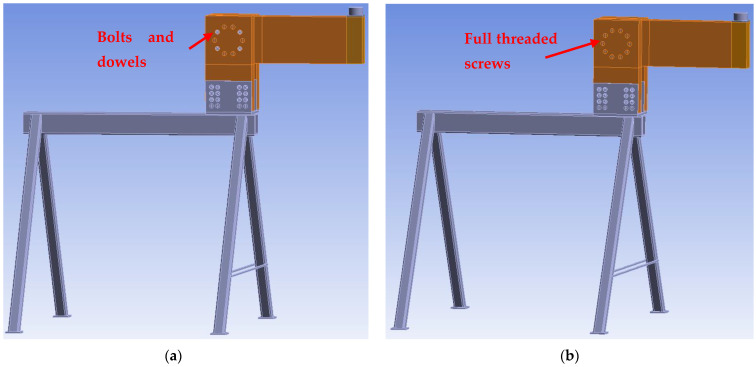

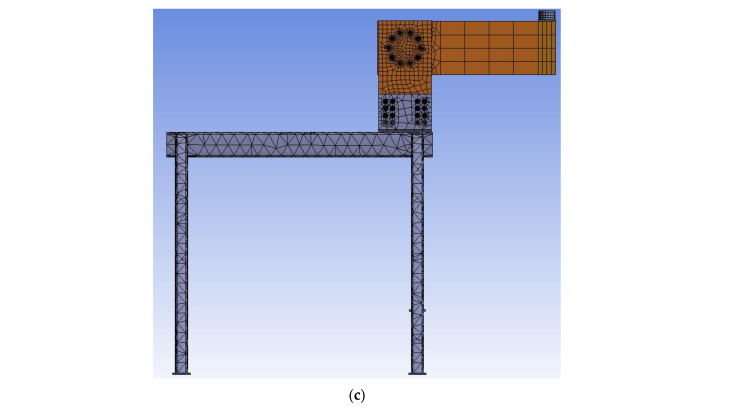
Numerical models: (**a**) Experiment A, bolts and dowels; (**b**) Experiment B, fully threaded screws; (**c**) finite element mesh in the overall model view.

Figure 7 shows individual numerical models that were used for the analysis of the load-carrying capacity. The connection with bolts and dowels (Experiment A) contained 253,961 nodes, 67,432 finite elements, and 730,642 equations. The connection with fully threaded screws (Experiment B) contained 237,743 nodes, 61,565 finite elements, and 678,822 equations.

**Figure 8:**
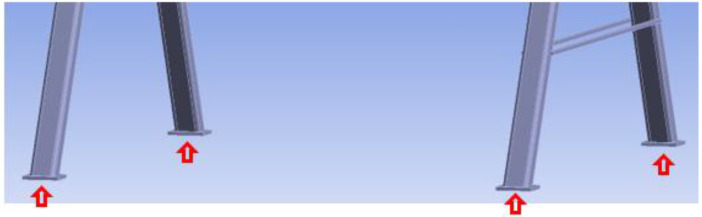
Boundary conditions of the numerical models.

Figure 8 shows the boundary conditions of the numerical model. A vertical displacement of all stands of the auxiliary steel structure was disabled. In addition, a vertical force of 5 kN was applied to each rear stand to prevent the vertical displacement and the overturning of the steel structure. Such a choice of boundary conditions ensured the real conditions that were used during the experimental testing.

**Figure 9:**
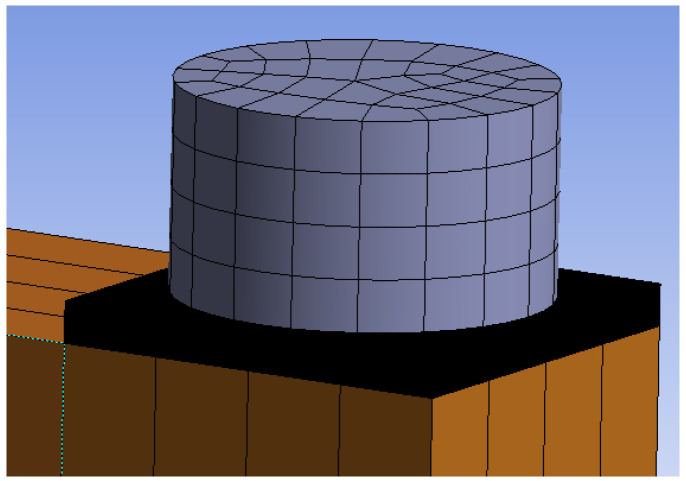
Finite element mesh for applying load.

It is very important to correctly apply the load into the numerical model. Figure 9 shows the finite element mesh for both numerical models for applying the load using the press head (cylinder) of the testing machine. The model was loaded as vertical displacement (displacement-controlled loading), which represented the actual loading by the cylinder during the experimental testing. The numerical model used the Newton–Raphson incremental–iterative method. The number of increments was the selected logic setting of the Ansys^TM^ software [47], with an initial step of 0.001 s and a maximum step of 0.04 s.

**Figure 10:**
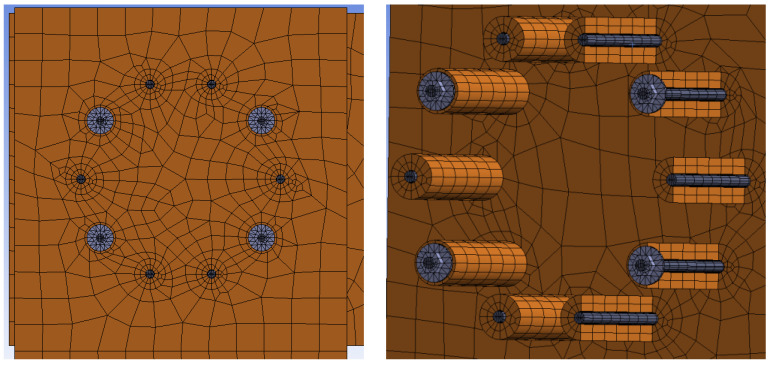
Finite element mech for Experiment A, bolts and dowels.

**Figure 11:**
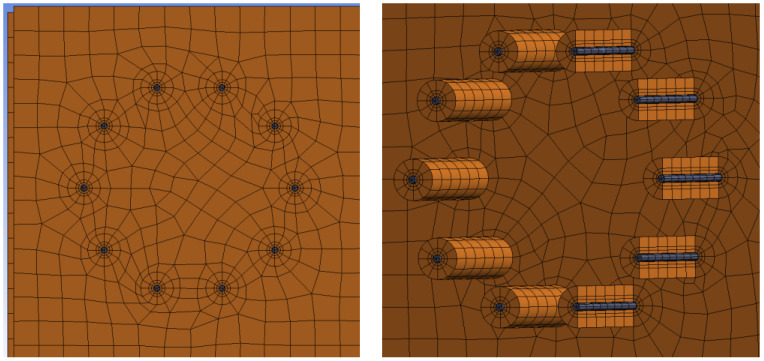
Finite element mech for Experiment B, fully threaded screws.

The finite element mesh was created from 3D finite elements. Tetrahedral finite elements were used for most of the steel structure, and hexahedral elements were used for the timber structure and fasteners. A finer mesh of finite elements was created around contact of timber with fasteners. The finite element ratio of 3:1 (length to height of finite elements) was used based on general FEM modeling recommendations; see *Brožovský 2012* [48]. Frictional contacts were used between the individual segments of the structure. The coefficient of friction between timber–timber elements was 0.40, steel–steel 0.10, and timber–steel 0.30 [51]. The interaction between the steel cylinder and the rubber pad was created using a coefficient of friction of 0.50. This rubber pad was firmly connected to the timber in order to achieve a good convergence. Figure 10 shows the finite element mesh for Experiment A, bolts and dowels. Figure 11 shows the finite element mesh for Experiment B, fully threaded screws.

## 3. Results

### 3.1. Results of Experimental Testing

#### 3.1.1. Experiment A, Bolts and Dowels

**Figure 12:**
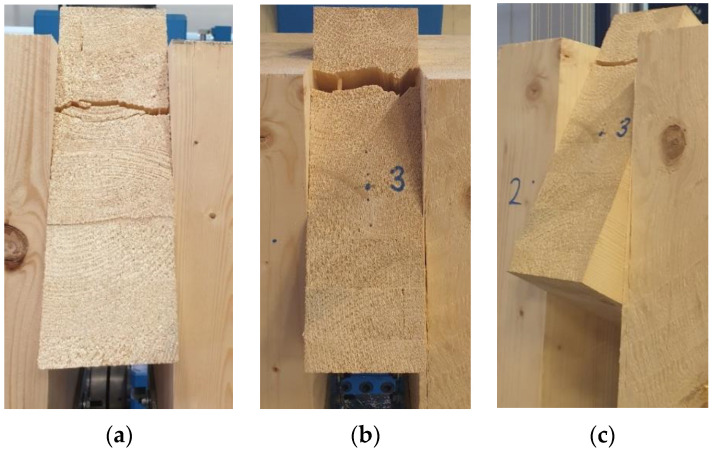
Experiment A, bolts and dowels: (**a**) test 1; (**b**) test 2; (**c**) test 3.

Figure 12 shows broken specimens after the quasi-static destructive experimental testing. Each specimen was broken in the rung when the tensile stress perpendicular to the grain was exceeded. It is important to state that the failure occurred in timber outside the glued joint between individual lamellas.

**Figure 13:**
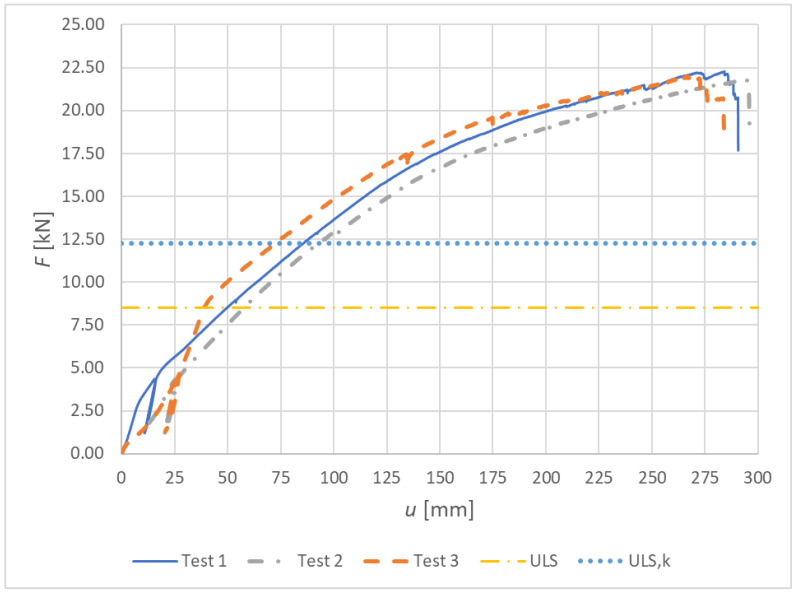
Experiment A: Load–deformation curves of destructive quasi-static cyclic testing for individual specimens made from a combination of bolts and dowels.

Figure 13 shows load–deformation curves from the experimental testing for Experiment A, bolts and dowels.

#### 3.1.2. Experiment B, Fully Threaded Screws

**Figure 14:**
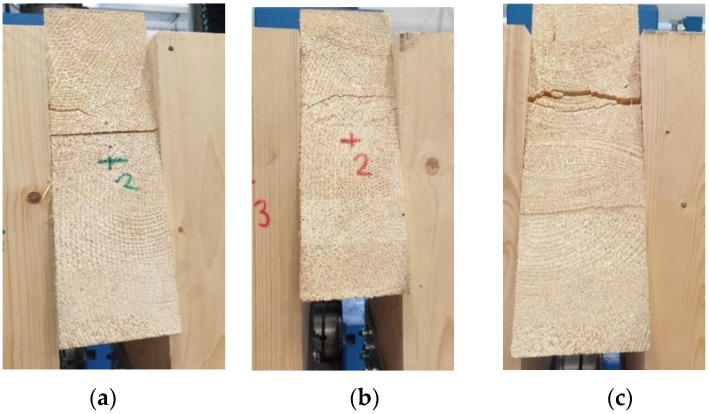
Experiment B, fully threaded screws: (**a**) test 1; (**b**) test 2; (**c**) test 3.

Figure 14 shows broken specimens after the quasi-static destructive experimental testing. Even when testing specimens with fully threaded screws, each specimen was broken in the rung when the tensile stress perpendicular to the grain was exceeded. The failure also occurred in timber outside the glued joint between individual lamellas.

**Figure 15:**
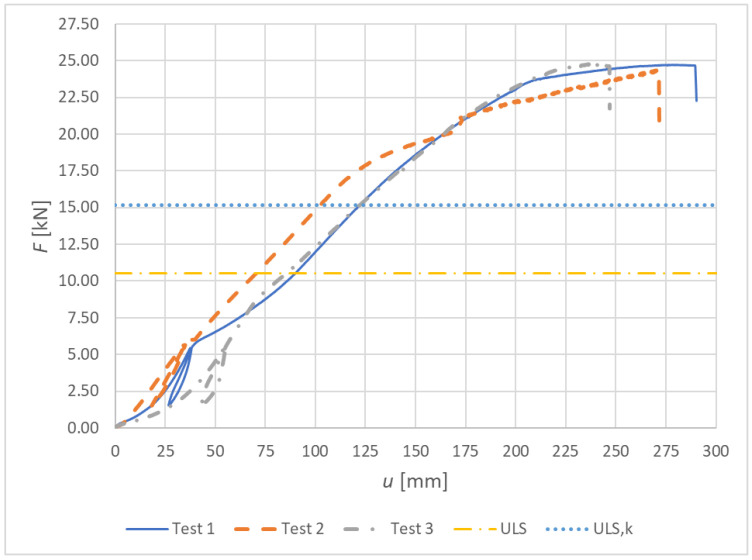
Experiment B: Load–deformation curves of destructive quasi-static cyclic testing for individual specimens made from fully threaded screws.

Figure 15 shows load–deformation curves from the experimental testing for Experiment B, fully threaded screws.

### 3.2. Tabular Results of the Experimental Testing

The results of the individual tests are listed in Table 6 and Table 7. The first column indicates the specimen number. The second column shows the maximum force achieved during loading (i.e., the actual load-carrying capacity). The third column shows the calculated load-carrying capacity according to *Eurocode 5* [20] (using modification factor *k_mod_* = 0.90 and partial factor for material properties *γ_con_* = 1.30). The fourth column contains the calculated load-carrying capacity without using the modification and partial factor. The fifth column shows the ratio between the load-carrying capacity based on the experimental testing and calculation according to *Eurocode 5* [20] using the modification and partial factor. The sixth column shows the ratio between the load-carrying capacity based on the experimental testing and calculation according to *Eurocode 5* [20] without using the modification and partial factor. The seventh column shows the maximum value of the vertical displacement of the rung end. The eighth column shows the maximum bending moment calculated from the value in the second column.

### 3.3. Results of Numerical Modeling

#### 3.3.1. Experiment A, Bolts and Dowels

**Figure 16:**
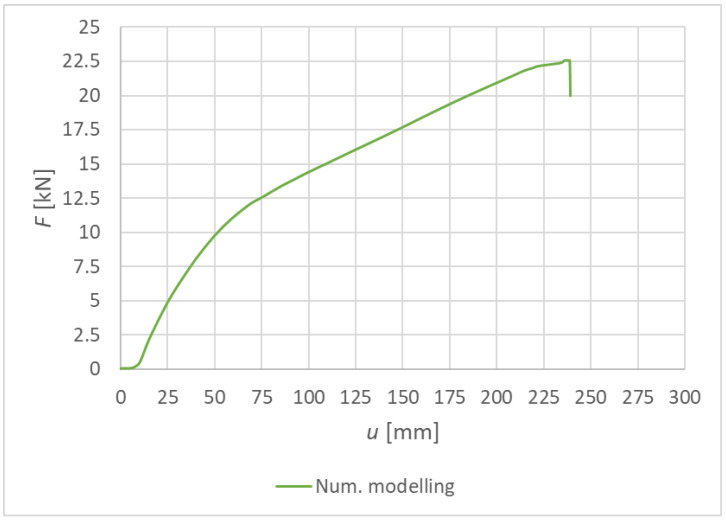
Experiment A: Load–deformation curves of quasi-static numerical modeling for individual specimens made from a combination of bolts and dowels.

Figure 16 shows the load–deformation curve from the numerical modeling for Experiment A, bolts and dowels. The values of the maximum achieved deformation and load-carrying capacity are shown in Table 8.

**Figure 17:**
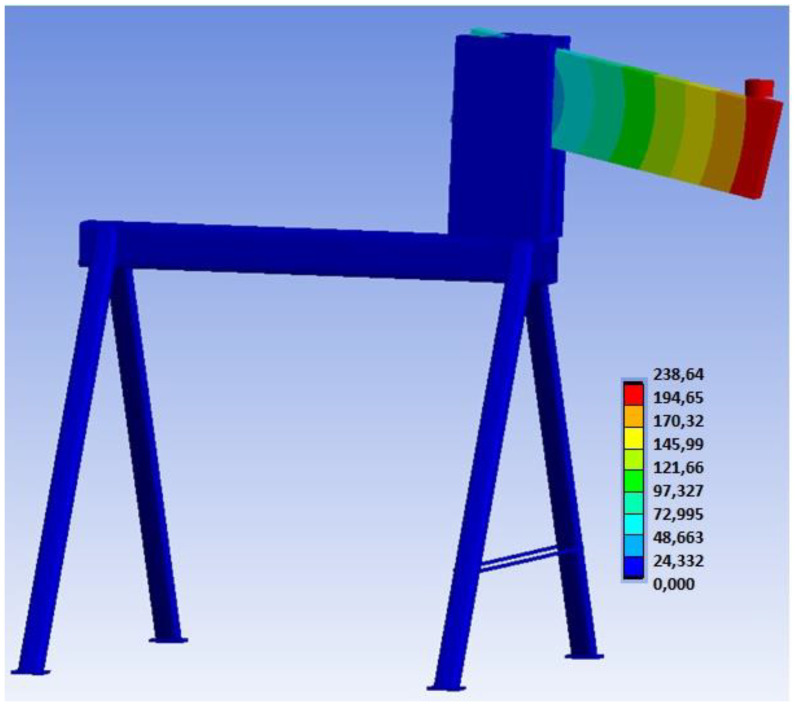
Overall deformation of the structure (mm) for Experiment A.

Figure 17 shows the overall deformation of the connection, including the deformation of the auxiliary steel structure. This deformation describes the actual behavior of the entire structure during loading.

**Figure 18:**
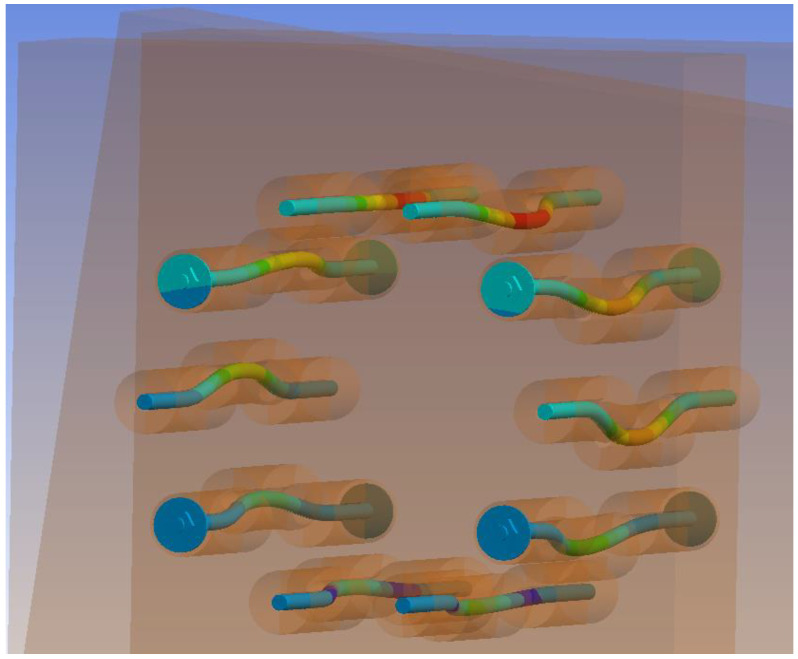
Overall deformation of fasteners (bolts and dowels) for Experiment A.

Figure 18 shows an “X-ray” view of the connection. It is possible to observe the embedment of timber around the holes for fasteners and the formation of two plastic hinges in fasteners.

**Figure 19:**
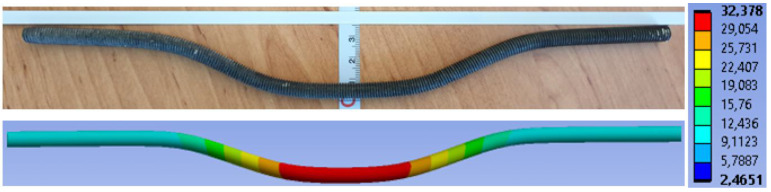
Deformation of a dowel (mm).

Figure 19 shows the overall deformation of a dowel. The deformation after the experimental testing with a value of 35.10 mm is above. The deformation from the numerical model with a value of 23.52 mm is below.

**Figure 20:**
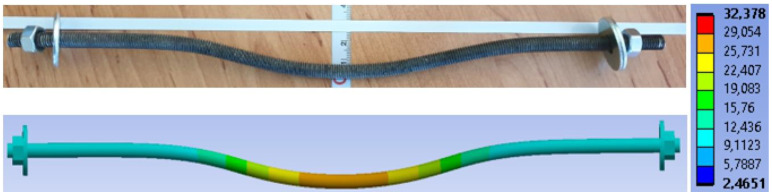
Deformation of a bolt (mm).

Figure 20 shows the overall deformation of a bolt. The deformation after the experimental testing with a value of 24.30 mm is above. The deformation from the numerical model with a value of 16.62 mm is below.

**Figure 21:**
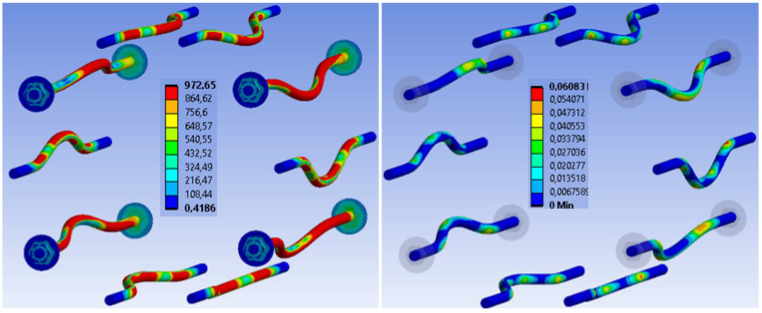
Experiment A: Stress in fasteners during connection collapse (MPa) (**left**), plastic deformation in fasteners during connection collapse (-) (**right**).

**Figure 22:**
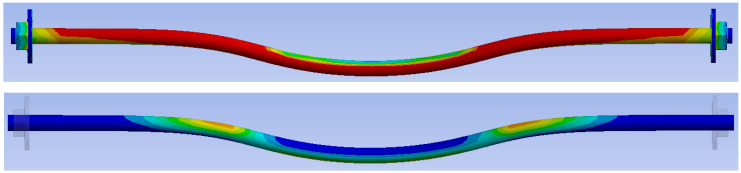
Experiment A: Detailed stress (MPa) (**above**), detailed plastic deformation (-) (**below**) (colour scale valid form the previous figure).

Figure 21 shows the stress and deformation in fasteners at a connection collapse in the numerical analysis. The stress during the collapse reaches the ultimate strength and the maximum plastic deformation reaches the value of 6.08%. On the basis of the experimental testing of used bolts and dowels (see the publication *Johanides 2022* [43]), it can be concluded that the numerical model did not fail due to the rupture of the fasteners. This fact was also verified and confirmed by the experimental testing of those connections. Figure 22 shows the detailed stress distribution and plastic deformation in one fastener.

**Figure 23:**
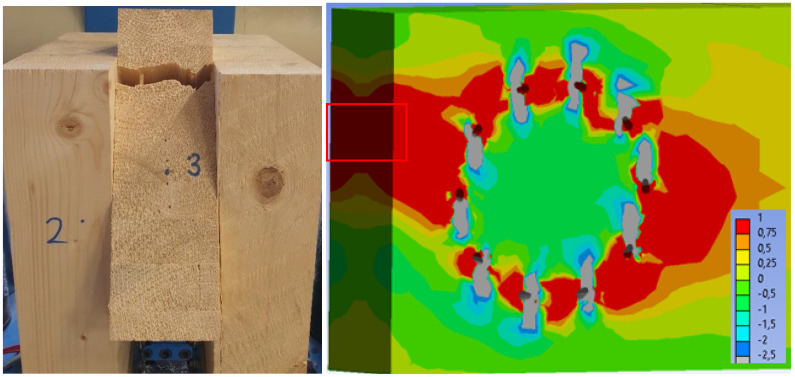
Experiment A: Collapse of the connection during experimental testing (on the (**left**)), tensile stress perpendicular to the grain from the numerical model (MPa) (on the (**right**)).

Figure 23 on the right shows the tensile stress perpendicular to the grain in the rung. When the numerical model collapses, the maximum stress (1 MPa) is found in a large area of timber. It can be especially observed in the critical area, which is marked in a red frame. Figure 23 on the left shows the collapse during the experimental testing. It can be seen that the failure occurred in the rung, precisely in the mentioned critical area. On the basis of these findings, it can be concluded that the numerical model failed because the tensile strength perpendicular to the grain in timber was exceeded.

**Figure 24:**
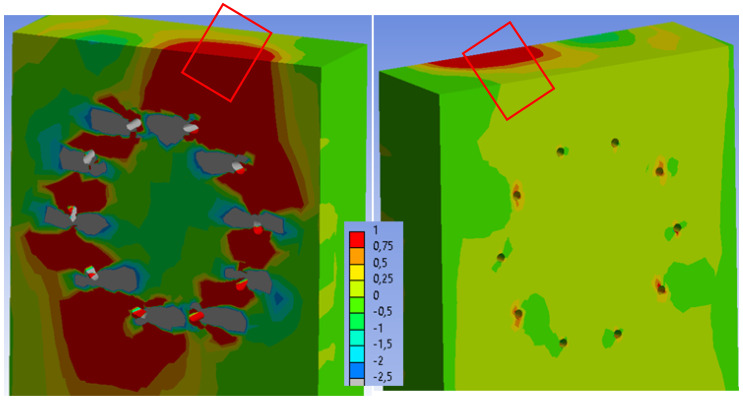
Experiment A: Collapse of the connection during experimental testing (on the (**left**)), tensile stress perpendicular to the grain from the numerical model (MPa) (on the (**right**)).

Figure 24 shows the tensile stress perpendicular to the grain in the stands. When the numerical model collapses, the maximum stress (1 MPa) is also found in a relatively large area of timber, but is not found through the entire cross-sectional area. Therefore, the second critical area of the connection (marked in Figure 24) could remain undamaged during the experimental testing. After exceeding the tensile stress perpendicular to the grain, the timber could be damaged from the inside of the connection with various cracks. However, this fact could not be detected during the experimental testing because this area was not visible.

#### 3.3.2. Experiment B, Fully Threaded Screws

**Figure 25:**
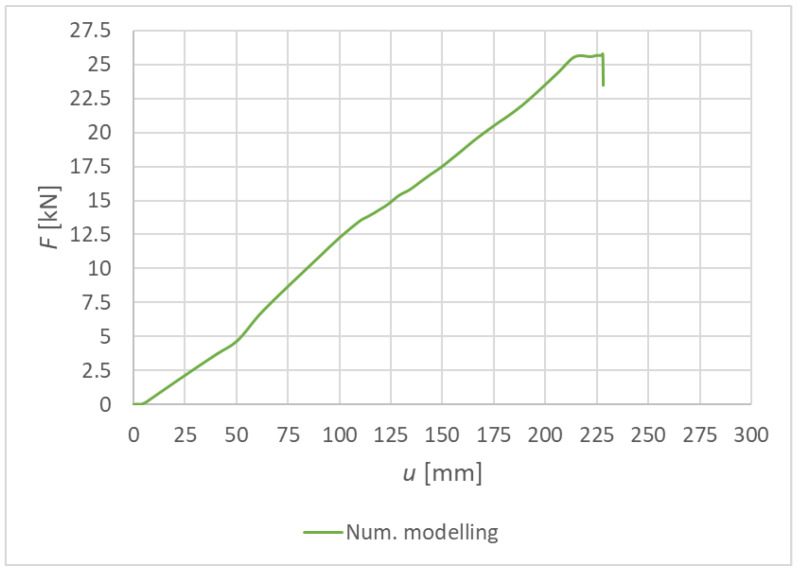
Experiment B: Load–deformation curves of quasi-static numerical modeling for individual specimens made from fully threaded screws.

Figure 25 shows the load–deformation curve from the numerical modeling for Experiment B, fully threaded screws. The values of the maximum achieved deformation and load-carrying capacity are shown in Table 9.

**Figure 26:**
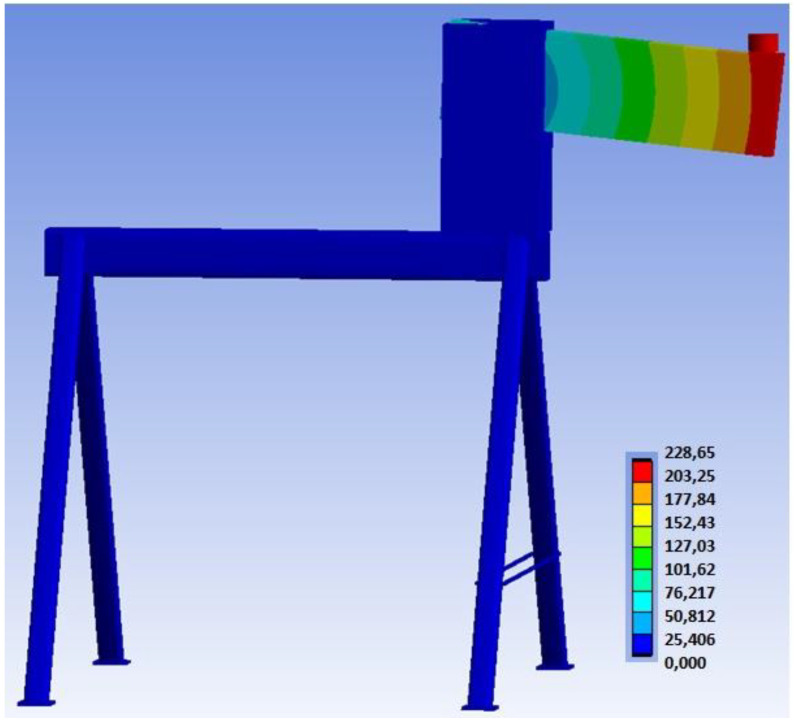
Overall deformation of the structure (mm) for Experiment B.

Figure 26 shows the overall deformation of the connection, including the deformation of the auxiliary steel structure. This deformation describes the actual behavior of the entire structure during loading.

**Figure 27:**
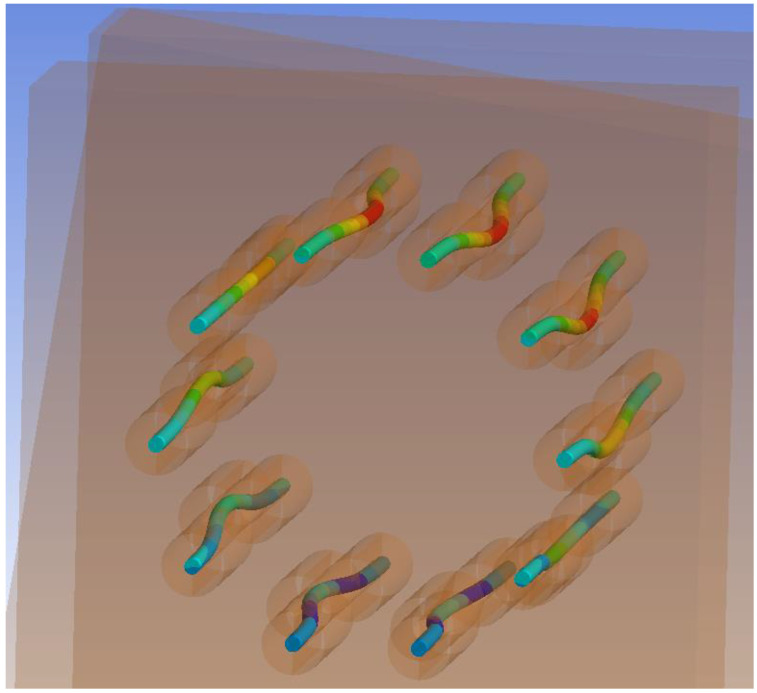
Overall deformation of fasteners (fully threaded screws) for Experiment B.

Figure 27 shows an “X-ray” view of the connection. It is possible to observe the embedment of timber around the holes for fasteners and the formation of two plastic hinges in fasteners.

**Figure 28:**
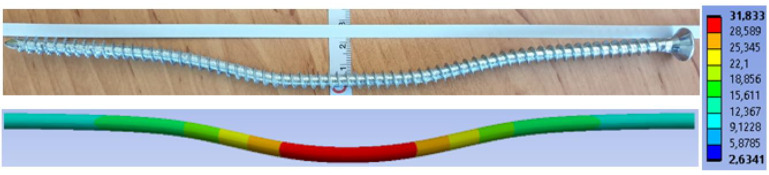
Deformation of a fully threaded screw (mm).

Figure 28 shows the overall deformation of a fully threaded screw. The deformation after the experimental testing with a value of 22.30 mm is above. The deformation from the numerical model with a value of 18.47 mm is below.

**Figure 29:**
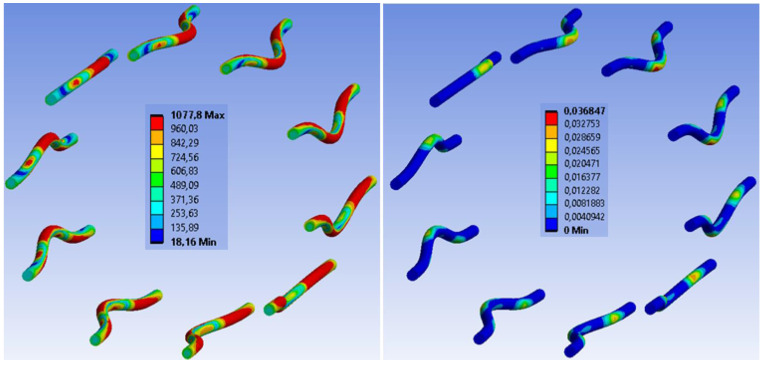
Experiment B: Stress in fasteners during connection collapse (MPa) (on the (**left**)), plastic deformation in fasteners during connection collapse (-) (on the (**right**).

**Figure 30:**
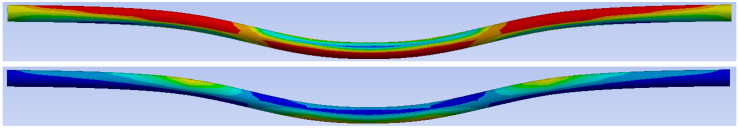
Experiment B: Detailed stress (MPa) (**above**), detailed plastic deformation (-) (**below**) (colour scale valid form the previous figure).

Figure 29 shows the stress and deformation in fasteners at a connection collapse in the numerical analysis. The stress during the collapse reaches the ultimate strength and the maximum plastic deformation reaches the value of 3.68%. On the basis of the experimental testing of used fully threaded screws (see the publication *Johanides 2022* [43]), it can be concluded that the numerical model did not fail due to the rupture of the fasteners. This fact was also verified and confirmed by the experimental testing of those connections. Figure 30 shows the detailed stress distribution and plastic deformation in one fastener.

**Figure 31:**
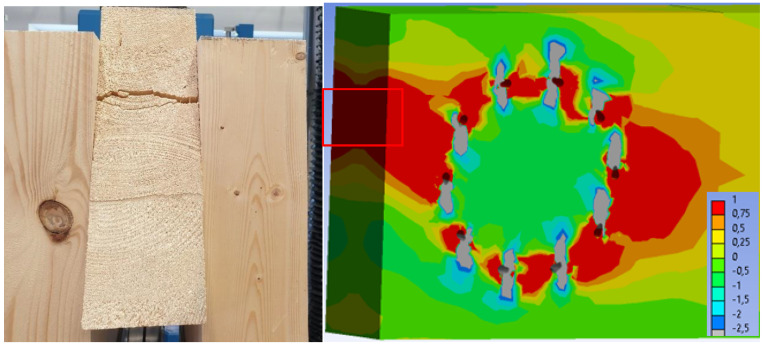
Experiment B: Collapse of the connection during experimental testing (on the (**left**)), tensile stress perpendicular to the grain from the numerical model (MPa) (on the (**right**)).

Figure 31 on the right shows the tensile stress perpendicular to the grain in the rung. When the numerical model collapses, the maximum stress (1 MPa) is found in a large area of timber. It can be especially observed in the critical area, which is marked in a red frame. Figure 31 on the left shows the collapse during the experimental testing. It can be seen that the failure occurred in the rung, precisely in the mentioned critical area. On the basis of these findings, it can be concluded that the numerical model failed because the tensile strength perpendicular to the grain in timber was exceeded.

**Figure 32:**
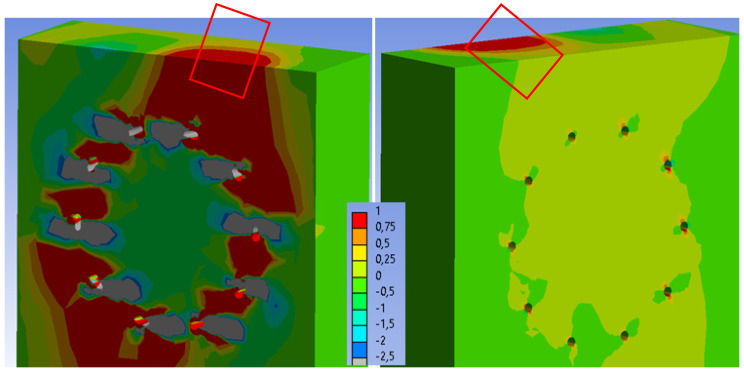
Experiment B: Collapse of the connection during experimental testing (on the (**left**)), tensile stress perpendicular to the grain from the numerical model (MPa) (on the (**right**)).

Figure 32 shows the tensile stress perpendicular to the grain in the stands. When the numerical model collapses, the maximum stress (1 MPa) is also found in a relatively large area of timber, but is not found through the entire cross-sectional area. Therefore, the second critical area of the connection (marked in Figure 32) could remain undamaged during the experimental testing.

## 4. Discussion

**Figure 33:**
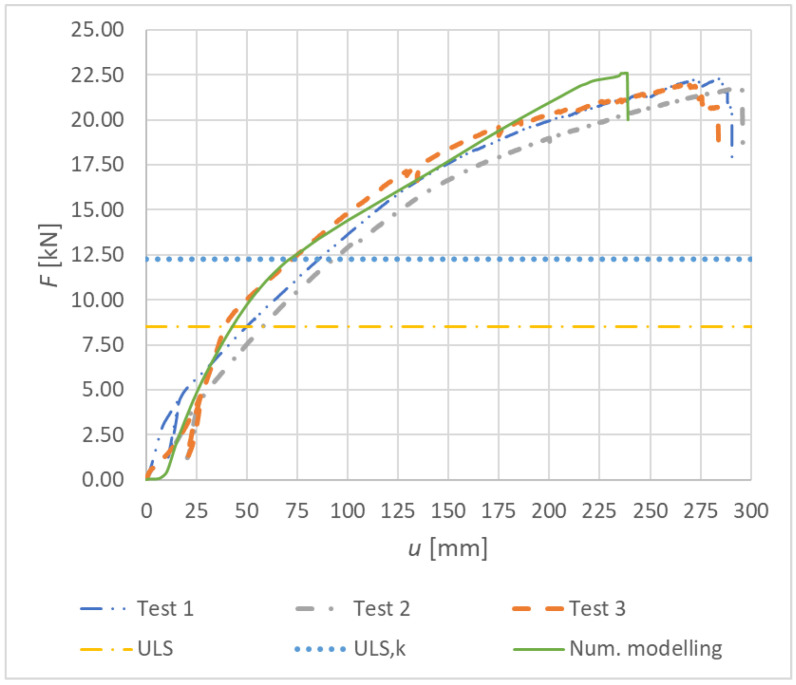
Load–deformation curves for Experiment A.

Figure 33 shows the load–deformation curves of Experiment A—numerical modeling (continuous curve) and experimental testing (dashed, dot-dashed, and double-dot-dashed curves). It is possible to observe a relatively good agreement of the real load-carrying capacities with the numerical analysis. The course of the numerical curve best matches the experimental test 3.

Results of the experimental testing and numerical modeling are given in Table 10. The table shows the maximum vertical deformation, the maximum force, and the corresponding bending moment at the point of connection failure. This value is compared with the value obtained from the numerical model. The maximum difference between the numerical value and the experimental value is 3.76%, and the minimum one is 1.40%. This agreement of the maximum load-carrying capacity can be considered as sufficiently accurate.

Less accuracy was achieved for the maximum deformation of the rung end. The maximum difference between the numerical model and the experimental test is 18.86%, and the minimum one is 15.84%. This inaccuracy could be caused by local imperfections in timber, local cracks in timber during loading, or the different modulus of the elasticity of the individual glued lamellas. These imperfections could not be taken into account in the numerical model.

**Figure 34:**
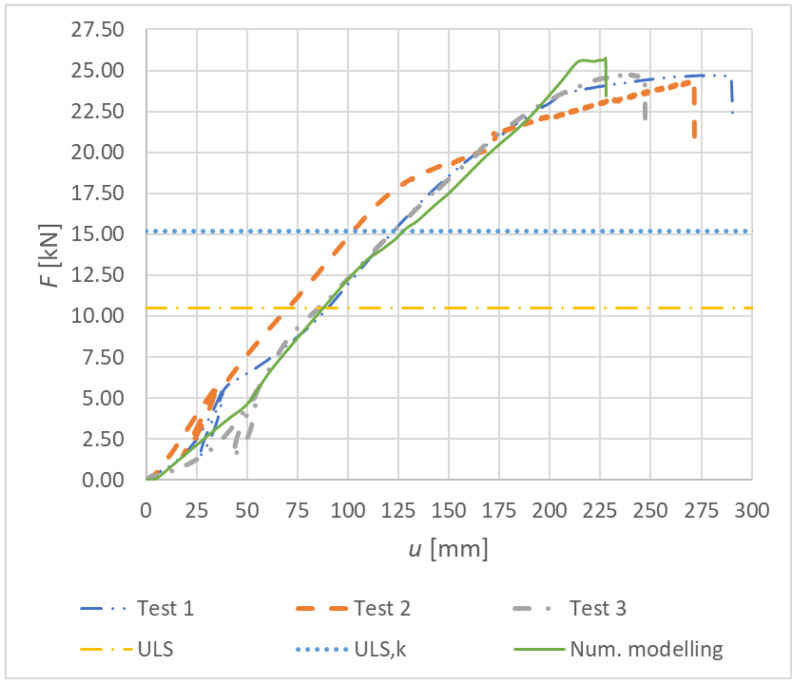
Load–deformation curves for Experiment B.

Figure 34 shows the load–deformation curves of Experiment B—numerical modeling (continuous curve) and experimental testing (dashed, dot-dashed, and double-dot-dashed curves). The course of the numerical curve best matches the experimental tests 1 and 3.

Results of the experimental testing and numerical modeling are given in Table 11. The table shows the maximum vertical deformation, the maximum force, and the corresponding bending moment at the point of connection failure. This value was compared with the value obtained from the numerical model. The maximum difference between the numerical value and the experimental value is 5.70%, and the minimum one is 4.20%. Although the achieved agreement of the maximum load-carrying capacity is worse compared to Experiment A (bolts and dowels), it can be considered as sufficiently accurate. Less accuracy was again achieved for the maximum deformation of the rung end. The maximum difference between the numerical model and the experimental test is 21.58%, and the minimum one is 7.81%. Even with great effort, it was not possible to achieve a better accuracy.

## 5. Conclusions

The paper was focused on the issue of the semi-rigid connection, which was composed of a timber rung and two stands using dowel-type fasteners. Six specimens were tested by quasi-static destructive cyclic loading. Three specimens were made from a combination of bolts and dowels and three specimens from fully threaded screws. The work required the creation of analytical assumptions, which were the basis for the design of the experimental testing and were subsequently used to compare the results.

First, a consolidation of the connection occurred during the initial phase of the loading. This phenomenon could be demonstrated on the load–deformation curve with a concave shape.

According to preliminary analytical and numerical calculations, the experimental testing of the real specimens resulted in an expected failure of connections in the area of the rung by tension perpendicular to the grain.

The actual measured load-carrying capacity of specimens made from a combination of bolts and dowels reached higher values compared to the design (ultimate limit state) load-carrying capacity calculated according to the standard. The actual load-carrying capacity was 2.54 times higher for the experimental test 1, 2.48 times higher for the experimental test 2, and 2.51 times higher for the experimental test 3.

The actual measured load-carrying capacity of specimens made from fully threaded screws also reached higher values compared to the design (ultimate limit state) load-carrying capacity calculated according to the standard. The actual load-carrying capacity was 2.08 times higher for the experimental test 1, 2.05 times higher for the experimental test 2, and 2.07 times higher for the experimental test 3.

It can be concluded that both types of the semi-rigid connection are sufficiently safe and reliable from the point of view of the load-carrying capacity.

Results of the numerical modeling were satisfactorily accurate in all cases compared to results of the experimental testing. Using the calibration of the numerical model according to the experimental data, it was possible to detect and describe stresses and deformations of the entire structure, as well as its details that are not visible at first sight during the experimental testing. Subsequently, it is possible to carry out analyses of different variations by removing or adding fasteners. This would save time, material, and money, because it would not be necessary to carry out further experimental testing.

The issue of determining the load-carrying capacity of connections in timber structures according to the European standards for the design of timber structures *Eurocode 5* [20] is still under development. The proposed experiments should also contribute to this trend. The experiments were focused on determining the load-carrying capacity of a semi-rigid connection of a rung and two stands using dowel-type mechanical fasteners. The data from the experiments can be used for the practical design of this type of a semi-rigid connection from the point of view of the load-carrying capacity.

## Figures and Tables

**Table 1:** The course of the experimental testing *Johanides 2022* [43].

Loading Step	Bolts and Dowels		Fully Threaded Screws	
	From (kN)	To (kN)	From (kN)	To (kN)
Step 1	0	4.90	0	6.08
Step 2	Hold		Hold	
Step 3	4.90	1.23	6.08	1.52
Step 4	Hold		Hold	
Step 5	1.23	8.58	1.52	10.63
Step 6	8.58	12.26	10.63	15.19

**Table 2:** Elastic constants of the material model for timber; see *Johanides 2022* [44].

Timber Properties	Value	Unit
Young’s modulus in *X*	9200	MPa
Young’s modulus in *Y*	740	MPa
Young’s modulus in *Z*	400	MPa
Poisson’s ratio in *XY*	0.47	-
Poisson’s ratio in *YZ*	0.25	-
Poisson’s ratio in *XZ*	0.37	-
Shear modulus in *XY*	650	MPa
Shear modulus in *YZ*	38	MPa
Shear modulus in *XZ*	700	MPa

**Table 3:** Elastic constants of the material model of fasteners; see *Johanides 2022* [44].

Steel Properties	Value	Unit
Young’s modulus	190,000	MPa
Poisson’s ratio	0.30	-

**Table 4:** Values for the plastic behavior of the material model for timber; *Johanides 2022* [44].

Hill Yield Criterion	Value	Unit
Yield strength in *X*	32	MPa
Yield strength in *Y*	1	MPa
Yield strength in *Z*	1	MPa
Yield strength in *XY*	6	MPa
Yield strength in *YZ*	3	MPa
Yield strength in *XZ*	6	MPa

**Table 5:** Values for the plastic behavior of the material model for steel; see *Johanides 2022* [44].

**Bolts and Dowels**		
	**Value**	**Unit**
Yield strength	670	MPa
Ultimate strength	970	MPa
Hardening modulus	1000	MPa
**Fully Threaded Screws**		
	**Value**	**Unit**
Yield strength	690	MPa
Ultimate strength	1075	MPa
Hardening modulus	1000	MPa

**Table 6:** Results of Experiment A, bolts and dowels; see *Johanides 2022* [43].

Specimen	*F*_max,test_ (kN)	*F*_max,d_ (kN)	*F*_max,k_ (kN)	*d* (-)	*k* (-)	*u* (mm)	*M* (kNm)
1	22.29	8.51	12.26	2.62	1.82	290.60	17.16
2	21.78			2.56	1.78	295.67	16.77
3	22.02			2.59	1.80	283.56	16.96

**Table 7:** Results of Experiment B, fully threaded screws; see *Johanides 2022* [43].

Specimen	*F*_max,test_ (kN)	*F*_max,d_ (kN)	*F*_max,k_ (kN)	*d* (-)	*k* (-)	*u* (mm)	*M* (kNm)
1	24.72	10.52	15.19	2.35	1.63	290.50	19.03
2	24.37			2.32	1.60	271.85	18.76
3	24.65			2.34	1.62	247.10	18.98

**Table 8:** Results of numerical modeling, Experiment A.

*F*_num_ (kN)	*u* (mm)	*M* (kNm)
22.60	238.64	17.40

**Table 9:** Results of numerical modeling, Experiment B.

*F*_num_ (kN)	*u* (mm)	*M* (kNm)
25.75	228.65	19.83

**Table 10:** Results of Experiment A (bolts and dowels).

Method	Specimen	*u* (mm)	*F* (kN)	*M* (kNm)	Difference *F* (%)	Difference *u* (%)
Numerical model	-	238.4	22.60	17.40	-	-
Experimental test	1	290.60	22.29	17.16	1.40	17.96
	2	295.67	21.78	16.77	3.76	19.37
	3	283.56	22.02	16.96	2.59	15.93

**Table 11:** Results of Experiment B (fully threaded screws).

Method	Specimen	*u* (mm)	*F* (kN)	*M* (kNm)	Difference *F* (%)	Difference *u* (%)
Numerical model	-	227.80	25.75	19.83	-	-
Experimental test	1	290.50	24.72	19.03	4.20	21.58
	2	271.85	24.37	18.76	5.70	16.20
	3	247.10	24.65	18.98	4.48	7.81

## Data Availability

Data are contained within the article.
